# Time series changes in pseudo-*R*^2^ values regarding maximum glomerular diameter and the Oxford MEST-C score in patients with IgA nephropathy: A long-term follow-up study

**DOI:** 10.1371/journal.pone.0232885

**Published:** 2020-05-07

**Authors:** Hiroshi Kataoka, Mamiko Ohara, Tomo Suzuki, Takahiro Inoue, Takafumi Akanuma, Keiko Kawachi, Shun Manabe, Yusuke Ushio, Kentaro Kawasoe, Taro Akihisa, Masayo Sato, Naomi Iwasa, Yukako Sawara, Kazuho Honda, Toshio Mochizuki, Ken Tsuchiya, Kosaku Nitta

**Affiliations:** 1 Department of Nephrology, Tokyo Women’s Medical University, Tokyo, Japan; 2 Clinical Research Division for Polycystic Kidney Disease, Department of Nephrology, Tokyo Women’s Medical University, Tokyo, Japan; 3 Department of Nephrology, Kameda Medical Center, Chiba, Japan; 4 Department of Anatomy, Showa University, Tokyo, Japan; 5 Department of Blood Purification, Kidney Center, Tokyo Women’s Medical University, Tokyo, Japan; University of Washington, UNITED STATES

## Abstract

There is no effectual pathological factor to predict the long-term renal prognosis of IgA nephropathy. Glomerular hypertrophy plays a crucial role in kidney disease outcomes in both experimental models and humans. This study aimed to 1) confirm the long-term prognostic significance of a maximal glomerular diameter (Max GD) ≥ 242.3 μm, 2) test a renal prognosis prediction model adding Max GD ≥ 242.3 μm to the Oxford classification (MEST-C), and 3) examine the time series changes in the long-term renal prognosis of patients with IgA nephropathy. The study included 43 patients diagnosed with IgA nephropathy from 1993 to 1998 at Kameda General Hospital. Renal prognosis with the endpoint of a 50% reduction in estimated glomerular filtration rate (eGFR) or the development of end-stage renal disease requiring dialysis was examined using logistic regression analysis, Cox regression analysis, and the Kaplan-Meier method. Pathological evaluation was performed using MEST-C and Max GD, and the validity of the prediction model was evaluated. Patients with Max GD ≥ 242.3 μm had significantly poor renal prognosis with multivariate Cox analysis (*P* = 0.0293). The results of the Kaplan-Meier analysis showed that kidney survival rates in the high-Max GD group were significantly lower than those in the low-Max GD group (log rank, *P* = 0.0043), which was confirmed in propensity score-matched models (log rank, *P* = 0.0426). Adding Max GD ≥ 242.3 μm to MEST-C improved diagnostic power of the renal prognosis prediction model by renal pathology tissue examination (*R*^2^: 3.3 to 14.5%, AICc: 71.8 to 68.0, C statistic: 0.657 to 0.772). We confirm that glomerular hypertrophy is useful as a long-term renal prognostic factor.

## Introduction

Although long-term renal prognoses of kidney diseases are clinically relevant, there is no established histopathological factor to predict the long-term renal prognosis of IgA nephropathy (IgAN). Knoop et al. used data from the Norwegian Kidney Biopsy Registry and found that none of the histopathological variables at the time of biopsy could identify patients with long-term progressive disease [1]. Coppo et al. conducted long-term follow-up analyses of the original Validation Study of the Oxford Classification for IgA Nephropathy cohort, and showed an independent relationship between kidney biopsy findings and the risk of progression towards kidney failure in patients with IgAN. However, in their prognostic analyses, inclusion of the entire set of pathology lesions provided only a slight gain (+1.8%) in discriminatory power over that of the clinical variables alone for the entire follow-up duration [2]. These results suggest that other factors are strongly associated with long-term disease progression of IgAN and that additional markers are clinically required to increase the prognostic efficacy of IgAN [3]. In truth, many studies on renal prognosis include not only histological factors but also clinical factors to predict renal outcome [4, 5]. In 2017, the presence of crescents (C) was added as a fifth parameter to the revised Oxford classification [6]. However, whether the MEST-C score quantitatively improves the long-term prediction of patients’ renal prognosis is not yet elucidated. We have previously reported that glomerular hypertrophy (or large renal corpuscles) with a maximum glomerular diameter (Max GD) of 242.3 μm or more as an aggravating factor for renal prognosis of IgAN patients observed for 10 years [7]. The aim of the present study is to examine the prediction efficacy a model in which Max GD ≥ 242.3 μm was added to the Oxford classification (MEST-C) in IgAN cases with long-term data on kidney disease progression.

## Subjects and methods

### Ethics statement

This research was approved by the ethics committee of Kameda Medical Center (No. 17–170), as with our previous study which used the same cohort [7]. After approval by the ethics committee, we used a passive informed consent (opt-out) for subjects. All data were analyzed anonymously.

### Patient selection

Between March 1993 and September 1998, 61 adult patients were diagnosed with IgAN based on their clinical profiles and renal biopsy findings at Kameda Medical Center. Among these, 3 were excluded due to estimated glomerular filtration rate (eGFR) < 50 mL/min/1.73 m^2^, 13 were excluded for a follow-up duration < 10 years, and 2 were excluded due to the presence of other renal disease. Finally, 43 patients were enrolled in the present study (S1 Fig). S1 Data include the measurement of covariates, definitions of comorbidities, and the histological assessment of kidney biopsies for all patients in the study.

### Pathological analysis

All kidney tissue specimens were obtained by percutaneous needle biopsy. Each specimen was evaluated for glomerular, interstitial, and vascular changes as previously described [8, 9]. The percentage of glomeruli that exhibited global sclerosis, segmental sclerosis, adhesions, and crescents were estimated. Mesangial cell proliferation, mesangial matrix expansion, interstitial fibrosis, interstitial inflammation, arteriosclerosis, and arteriolar hyalinosis in each patient were semi-quantitatively scored. The Oxford MEST-C criteria [6, 9, 10] was assessed by the following parameters: mesangial hypercellularity (M0: < 50% of glomeruli showing hypercellularity; M1: > 50% of glomeruli showing hypercellularity), endocapillary hypercellularity (E0: absent; E1: present), segmental glomerulosclerosis (S0: absent; S1: present), tubular atrophy/interstitial fibrosis (T0: < 25%; T1: 25–50%; T2: > 50% of cortical area involved), and cellular/fibrocellular crescents (C0: absent; C1: present in a least 1 but <25% of glomeruli; C2: present in at least 25% of glomeruli). We also assessed the maximal glomerular area (Max GA) and the Max GD of the maximally-hypertrophied glomerulus (the largest renal corpuscle) identified in serial sections [7]. Max GD was calculated as the mean of two measurements, i.e., the maximal diameter of the maximal profile area in the largest renal corpuscle [11], and the maximal chord perpendicular to the maximal diameter in each specimen [7].

### Study outcome

The primary outcome of the study was kidney disease progression, defined as a ≥ 50% decline in the eGFR from baseline (≥ 50% eGFR decline), or the development of end-stage renal disease (ESRD) requiring dialysis. The patients were followed up until November 2017.

### Statistical analysis

Continuous variables are reported as means and standard deviations, or as medians (minimum–maximum). Categorical variables are reported as percentages, unless otherwise stated. Group differences were evaluated using the unpaired *t*-test, Mann-Whitney U test, Chi-square test, or Fisher’s exact test, as appropriate. Logistic regression analyses were used to assess the discriminatory ability and goodness of fit of renal prognostic models. Renal prognostic factors were also evaluated in Cox regression analyses, and the Kaplan-Meier method was used for survival analyses. The prognostic variables for the renal outcomes were assessed using univariate and multivariate Cox proportional hazards models. Variables with *P* values < 0.1 in the univariate model, as well as age, sex, and eGFR, were included in the multivariate model. Based on the previous report [8], we divided the patients into 2 groups consisting of the high-Max GD group (Max GD ≥ 242.3 μm) and low-Max GD group (Max GD < 242.3 μm). Survival curves were plotted using the Kaplan-Meier method and evaluated using the log-rank test. To reduce confounding biases, we fitted propensity score-matched models that included potentially modifying variables, namely, age and eGFR; additionally, subgroup analyses were performed. The caliper-matching method was used, with a maximum tolerance level of 0.1.

To evaluate the Oxford classification, components of the MEST-C score with and without large renal corpuscles (Max GD ≥ 242.3 μm) were considered. The discriminatory ability of the model was evaluated using the concordance (C)-statistic [12, 13]. Goodness of fit was assessed using McFadden’s pseudo-*R*-squared (pseudo-*R*^2^) [14] and the corrected Akaike Information Criterion (AICc) [15]. The statistical tests were 2-tailed, and *P* < 0.05 was considered statistically significant. Statistical analyses were performed using JMP Pro software, version 14.1.0 (SAS Institute, Cary, NC, USA).

## Results

### Patients

The 43 patients included 25 men and 28 women, and their mean age at the time of the renal biopsy was 39.8 ± 9.8 years. The average value of MBP was 102.6 ± 16.8 mmHg, BMI was 25.4 ± 4.3 kg/m^2^, proteinuria was 1.4 (minimum–maximum, 0–7.0) g/day, and eGFR was 78.6 ± 17.5 mL/min/1.73 m^2^. Of the 43 patients, 22 had been treated with a corticosteroid and 25 with either taking an angiotensin-converting enzyme inhibitor or an angiotensin receptor blocker during the follow-up period. The median duration of follow-up was 14.4 (3.8–24.2) years, with 23 patients reaching the primary outcome (ESRD, n = 16; ≥ 50% eGFR decline, n = 21).

### Histopathological findings

The average number of glomeruli examined per patient was 13.4 ± 5.5 and the average number of serial sections examined per patient was 14.4 ± 3.2. The global glomerulosclerosis rate was 14.3% (0%– 57.1%). The average Max GD was 221.7 ± 30.8 μm. The median rate of global sclerosis, segmental sclerosis, crescent, and interstitial fibrosis were 14.3%, 14.7%, 6.3%, and 5.0%, respectively (Table 1B). The percentages of patients with regards Oxford classification variables were 81.4%, 53.5%, 81.4%, 2.3%, 2.3%, 46.5%, and 9.3% for M1, E1, S1, T1, T2, C1, and C2, respectively (Table 1B).

**Table 1:** Patient characteristics according to baseline Max GD levels.

**(Table 1A. Clinical and laboratory findings; Entire cohort, n = 43)**					
Variables	Entire cohort	Max GD ≥ 242.3 μm	Max GD < 242.3 μm	*P*-value	Standardized Differences
	n = 43	n = 15	n = 28		
*Clinical Findings*					
Age (years)	39.8 ± 9.8 [43]	41.4 ± 9.5	39.0 ± 10.0	0.4539	0.246
Sex (Men; n (%))	26 (60.5) [43]	11 (73.3)	15 (53.6)	0.3274	0.418
BMI (kg/m^2^)	25.4 ± 4.3 [43]	28.5 ± 4.2	23.7 ± 3.4	0.0003	1.256
MBP (mmHg)	102.6 ± 16.8 [43]	104.8 ± 15.9	101.4 ± 17.5	0.5417	0.203
*Laboratory Findings*					
Total Protein (g/dL)	6.48 ± 0.59 [43]	6.71 ± 0.51	6.36 ± 0.59	0.0566	0.635
Serum Albumin (g/dL)	3.79 ± 0.39 [43]	3.91 ± 0.40	3.73 ± 0.38	0.1487	0.461
Blood Urea Nitrogen (mg/dL)	14.5 ± 3.4 [43]	15.6 ± 2.7	13.9 ± 3.6	0.1127	0.534
Serum Creatinine (mg/dL)	0.83 ± 0.20 [43]	0.91 ± 0.22	0.79 ± 0.19	0.0719	0.584
eGFR (mL/min/1.73m^2^)	78.6 ± 17.5 [43]	72.2 ± 12.9	81.9 ± 18.9	0.0842	0.599
Uric Acid (mg/dL)	5.74 ± 1.58 [43]	6.01 ± 1.56	5.60 ± 1.60	0.4210	0.259
Total Cholesterol (mg/dL)	195.8 ± 44.3 [43]	197.3 ± 20.0	194.9 ± 53.3	0.8677	0.060
Triglyceride (mg/dL)	164.3 ± 134.2 [43]	228.7 ± 201.3	129.8 ± 58.9	0.0193	0.667
Hemoglobin A1c (NGSP) (%)	5.43 ± 0.94 [30]	6.08 ± 1.32	5.11 ± 0.44	0.0052	0.986
IgG (mg/dL)	1141.8 ± 321.0 [43]	1173.8 ± 401.4	1124.6 ± 275.4	0.6376	0.143
IgA (mg/dL)	337.1 ± 139.7 [43]	362.1 ± 162.2	323.7 ± 127.2	0.3971	0.263
IgM (mg/dL)	166.0 ± 92.0 [43]	160.6 ± 84.3	168.9 ± 97.3	0.7832	0.091
CH50 (mg/dL)	40.8 ± 5.9 [34]	40.4 ± 5.5	41.0 ± 6.3	0.7791	0.101
C3 (mg/dL)	90.4 ± 19.2 [42]	103.6 ± 21.6	83.9 ± 14.2	0.0010	1.078
C4 (mg/dL)	36.9 ± 10.9 [42]	38.7 ± 11.3	36.0 ± 10.8	0.4590	0.244
IgA/C3 ratio	3.85 ± 1.81 [42]	3.52 ± 1.64	4.01 ± 1.90	0.4158	0.276
U-Prot (g/day)	1.4 (0.0–7.0) [43]	1.5 (0.6–5.7)	1.1 (0.0–7.0)	0.5493	0.028
U-RBC (counts/HPF)	10 (0–200) [41]	1 (0–100)	10 (0–200)	0.0828	0.386
*Initial treatments*					
Corticosteroids (n (%))	22 (51.2) [43]	6 (40.0)	16 (57.1)	0.3475	0.347
Tonsillectomy (n (%))	2 (4.7) [43]	0 (0.0)	2 (7.1)	0.5349	0.391
Immunosuppressants (n (%))	0 (0.0) [43]	0 (0.0)	0 (0.0)	NA	NA
*Concomitant drugs*					
Antihypertensive agents (n (%))	30 (69.8) [43]	11 (73.3)	19 (67.9)	1.0000	0.119
ARB and or ACEI (n (%))	25 (58.1) [43]	9 (60.0)	16 (57.1)	1.0000	0.059
Others (n (%))	14 (32.6) [43]	7 (46.7)	7 (25.0)	0.1837	0.465
Anti-platelet agents	42 (97.7) [43]	14 (93.3)	28 (100.0)	0.3488	0.379
Anti-coagulation	11 (25.6) [43]	3 (20.0)	8 (28.6)	0.7190	0.202
Antidyslipidemic agents (n (%))	1 (2.3) [43]	1 (6.7)	0 (0.0)	0.3488	0.379
Antihyperuricemic agents (n (%))	5 (11.6) [43]	2 (13.3)	3 (10.7)	1.0000	0.080
*Comorbidities*					
Hypertension (n (%))	33 (76.7) [43]	12 (80.0)	21 (75.0)	1.0000	0.120
Hyperuricemia (n (%))	12 (27.9) [43]	7 (46.7)	5 (17.9)	0.0739	0.647
Hypertriglyceridemia (n (%))	16 (37.2) [43]	8 (53.3)	8 (28.6)	0.1849	0.519
Hypercholesterolemia (n (%))	10 (23.3) [43]	3 (20.0)	7 (25.0)	1.0000	0.120
**(Table 1B. Histological findings; Entire cohort, n = 43)**					
Variables	Entire cohort	Max GD ≥ 242.3 μm	Max GD < 242.3 μm	*P*-value	Standardized Differences
	n = 43	n = 15	n = 28		
Number of glomeruli	13.4 ± 5.5 [43]	12.1 ± 4.5	14.1 ± 5.9	0.2767	0.381
Global sclerosis (%)	14.3 (0.0–57.1) [43]	12.5 (0.0–55.6)	14.5 (0.0–57.1)	0.9693	0.037
Segmental sclerosis (%)	14.7 (0.0–69.2) [43]	11.1 (0.0–55.6)	17.7 (0.0–69.2)	0.2460	0.277
Crescent (%)	6.3 (0.0–83.3) [43]	6.3 (0.0–33.3)	7.4 (0.0–83.3)	0.6312	0.232
Cellular or Fibro-Cellular (%)	6.3 (0.0–83.3) [43]	6.3 (0.0–22.2)	6.1 (0.0–83.3)	0.5938	0.297
Fibrous (%)	0.0 (0.0–12.5) [43]	0.0 (0.0–12.5)	0.0 (0.0–8.3)	0.3101	0.410
Mesangial cell proliferation (0–3)	2 (1–3) [43]	3 (1–3)	2 (1–3)	0.1908	0.428
Interstitial fibrosis (%)	5.0 (0.0–60.0) [43]	5.0 (0.0–60.0)	5.0 (0.0–30.0)	0.7766	0.174
Interstitial fibrosis (0–3)	1 (0–2) [43]	1 (0–2)	1 (0–2)	0.8161	0.098
Interstitial inflammation (%)	5.0 (0.0–30.0) [43]	5.0 (0.0–25.0)	5.0 (0.0–30.0)	0.5248	0.277
Arteriosclerosis (0–3)	1 (0–2) [43]	1 (0–2)	0 (0–2)	0.0082	0.878
Arteriolar hyalinosis (0–3)	1 (0–3) [43]	1 (0–3)	1 (0–2)	0.0017	1.132
Max GD (μm)	221.7 ± 30.8 [43]	253.1 ± 10.9	204.9 ± 24.0	<0.0001	2.586
Max GA (μm)	37333.8 ± 10230.7 [43]	48024.0 ± 5034.0	31606.9 ± 7223.2	<0.0001	2.637
*Oxford Classification*					
M1	35 (81.4) [43]	12 (80.0)	23 (82.1)	1.0000	0.054
E1	23 (53.5) [43]	9 (60.0)	14 (50.0)	0.7492	0.202
S1	35 (81.4) [43]	11 (73.3)	24 (85.7)	0.4188	0.311
T1	1 (2.3) [43]	0 (0.0)	1 (3.6)	1.0000	0.273
T2	1 (2.3) [43]	1 (6.7)	0 (0.0)	0.3488	0.379
C1	20 (46.5) [43]	8 (53.3)	12 (42.9)	0.5401	0.209
C2	4 (9.3) [43]	0 (0.0)	4 (14.3)	0.2802	0.578

Continuous variables are expressed as means ± standard deviation or median (minimum–maximum). Count data are expressed as n (%). For each variable, the number of patients with non-missing data is shown in [].

Abbreviations: n, number; %, percentages; BMI, body mass index; MBP, mean blood pressure; GFR, estimated glomerular filtration rate; NGSP, national glycohemoglobin standardization program; IgG, immunoglobulin G; IgA, immunoglobulin A; IgM, immunoglobulin M; CH50, 50% hemolytic complement activity; C3, complement component 3; C4, complement component 4; U-Prot, Urinary protein excretion; U-RBC, urinary red blood cells; HPF, high-power field; ARB, angiotensin receptor blocker; ACEI, angiotensin-converting enzyme inhibitor; Max GD, maximal glomerular diameter; Max GA, maximal glomerular area; M, mesangial hypercellularity; E, endocapillary hypercellularity; S, segmental glomerulosclerosis; T, tubular atrophy/interstitial fibrosis; C, cellular/fibrocellular crescents

### High-Max GD relationship to long-term renal outcomes

**Fig 1:**
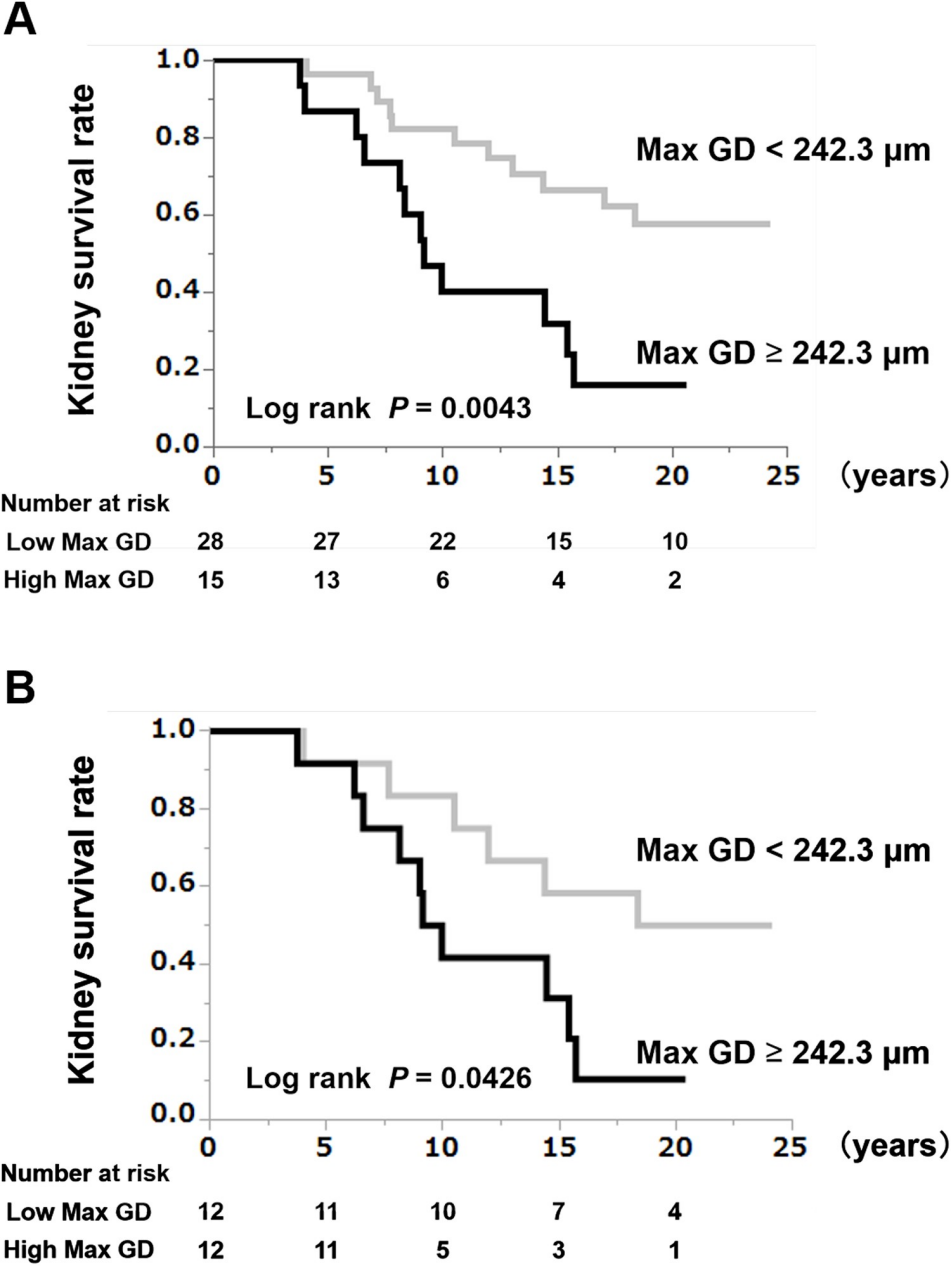
(A) Kidney survival rates in the high-Max GD group (Max GD ≥ 242.3 μm) and low-Max GD group (Max GD < 242.3 μm) within the entire cohort. The renal prognosis for patients with glomerular hypertrophy with a Max GD ≥ 242.3 μm was poor. Max GD: maximal glomerular diameter. (B) Kidney survival rate in the high-Max GD group (Max GD ≥ 242.3 μm) and low-Max GD group (Max GD < 242.3 μm) in the propensity score-matched cohort. The renal prognosis for patients with glomerular hypertrophy and a Max GD ≥ 242.3 μm was poor after matching the groups for age and eGFR. Max GD: maximal glomerular diameter; eGFR, estimated glomerular filtration rate.

To examine whether Max GD has relationship with long term renal function decline, we performed univariate and multivariate Cox regression analyses to detect any associations between the baseline clinical and histopathological findings and a ≥ 50% eGFR decline or ESRD during the follow-up period (Table 2). The multivariate Cox regression analyses revealed a significant association between the primary outcome (≥ 50% eGFR decline or ESRD) and a Max GD ≥242.3 μm (HR: 3.08, 95% confidence interval [CI]: 1.12–8.69; *P* = 0.0293). Kaplan-Meier analysis showed that the kidney survival rate in the high-Max GD group was significantly lower than that in the low-Max GD group (log rank, *P* = 0.0043) (Fig 1A).

**Table 2:** Univariate and multivariate analysis of risk factors associated with a ≥ 50% eGFR decline or ESRD (Entire cohort, n = 43).

Variables	Univariate analysis		Multivariate analysis	
	Hazard Ratio (95% CI)	*P*-value	Hazard Ratio (95% CI)	*P*-Value
*Clinical and Laboratory Findings*				
Age (1 year increase)	1.02 (0.98–1.07)	0.3017	0.97 (0.91–1.05)	0.5115
Men (vs. women)	1.95 (0.83–5.07)	0.1283	1.62 (0.45–6.05)	0.4513
BMI (1 kg/m^2^ increase)	1.08 (0.97–1.18)	0.1449	-	-
MBP (1 mmHg increase)	1.00 (0.98–1.03)	0.8917	-	-
eGFR (1 mL/min/1.73 m^2^ increase)	0.93 (0.89–0.96)	<0.0001	0.94 (0.89–0.98)	0.0082
Hemoglobin (1 g/dL increase)	0.96 (0.78–1.17)	0.6879	-	-
Serum albumin (1 g/dL increase)	1.02 (0.637–3.08)	0.9653	-	-
U-Prot (g/day)	1.00 (0.74–1.27)	0.9797	-	-
Hypercholesterolemia (vs. no)	0.58 (0.17–1.55)	0.2950	-	-
Hypertriglyceridemia (vs. no)	1.07 (0.44–2.46)	0.8699	-	-
Hyperuricemia (vs. no)	3.41 (1.44–7.84)	0.0062	0.76 (0.21–2.76)	0.6661
*Initial treatments*				
Corticosteroids (vs. no)	0.47 (0.20–1.09)	0.0789	0.65 (0.22–1.82)	0.4136
Tonsillectomy (vs. no)	2.24 (0.36–7.72)	0.3289	-	-
*Histological findings*				
Global sclerosis (%)	1.01 (0.99–1.04)	0.3190	-	-
Segmental sclerosis (%)	0.99 (0.96–1.02)	0.4142	-	-
Crescent (%)	1.00 (0.97–1.03)	0.7976	-	-
Mesangial cell proliferation (0–3)	1.26 (0.54–2.86)	0.5855	-	-
Interstitial fibrosis (0–3)	3.77 (1.53–8.55)	0.0049	2.26 (0.79–6.58)	0.1240
Interstitial inflammation (0–3)	1.64 (0.77–3.52)	0.2021	-	-
Arteriosclerosis (0–3)	1.10 (0.63–1.87)	0.7247	-	-
Arteriolar hyalinosis (0–3)	2.22 (1.26–3.82)	0.0062	1.02 (0.47–2.25)	0.9551
Max GD (1 μm increase)	1.02 (1.01–1.04)	0.0061	NA	-
Max GD ≥242.3 μm (vs. not)	3.16 (1.37–7.38)	0.0076	3.08 (1.12–8.69)	0.0293
*Oxford Classification*				
M0/M1	1.64 (0.56–6.95)	0.3977	NA	-
E0/E1	1.28 (0.56–2.99)	0.5618	NA	-
S0/S1	1.22 (0.46–4.23)	0.7077	NA	-
T0/T1/T2	2.07 (0.36–6.28)	0.3475	NA	-
C0/C1/C2	0.96 (0.50–1.75)	0.8960	NA	-

Variables with *P*-values of less than 0.1 in the univariate model, age, sex, and eGFR were included in the multivariate model.

Abbreviation: eGFR, estimated glomerular filtration rate; ESRD, end-stage renal disease; n, number; %, percentages; CI = confidence interval; vs, versus; BMI, body mass index; MBP, mean blood pressure; U-Prot, Urinary protein excretion; Max GD, maximal glomerular diameter; NA, not applicable; M, mesangial hypercellularity; E, endocapillary hypercellularity; S, segmental glomerulosclerosis; T, tubular atrophy/interstitial fibrosis; C, cellular/fibrocellular crescents

### Comparison of clinical and pathological findings between high- and low-Max GD groups

Comparative analyses revealed that, in the high-Max GD group, the levels of serum creatinine, triglyceride, hemoglobin A1c, and C3c as well as BMI were higher than those in the low-Max GD group (Table 1A). In histological findings, the level of arteriosclerosis and arteriolar hyalinosis were more severe in the high-Max GD group (Table 1B).

Since Max GD was associated with age and eGFR, we fit propensity score-matched models that included potential modifying variables (age and eGFR) and performed subgroup analyses of both groups. Comparative analyses in a propensity score-matched cohort revealed that there were no significant differences between the high-Max GD group and the low-Max GD group in any of the parameters except those associated with Max GD and Max GA levels (high-Max GD group vs. low-Max GD group, 253.9 ± 11.9 μm vs. 218.2 ± 16.2 μm; *P* < 0.0001) (S1 Table). The results of the Kaplan-Meier analysis with a ≥ 50% eGFR decline or ESRD as the end-point showed that the kidney survival rate of the high-Max GD of IgAN patients was significantly lower than the low-Max GD group, even after adjustment for the age and eGFR (log rank, *P* = 0.0426) (Fig 1B).

### Prognostic value of Max GD over study follow-up times

**Fig 2:**
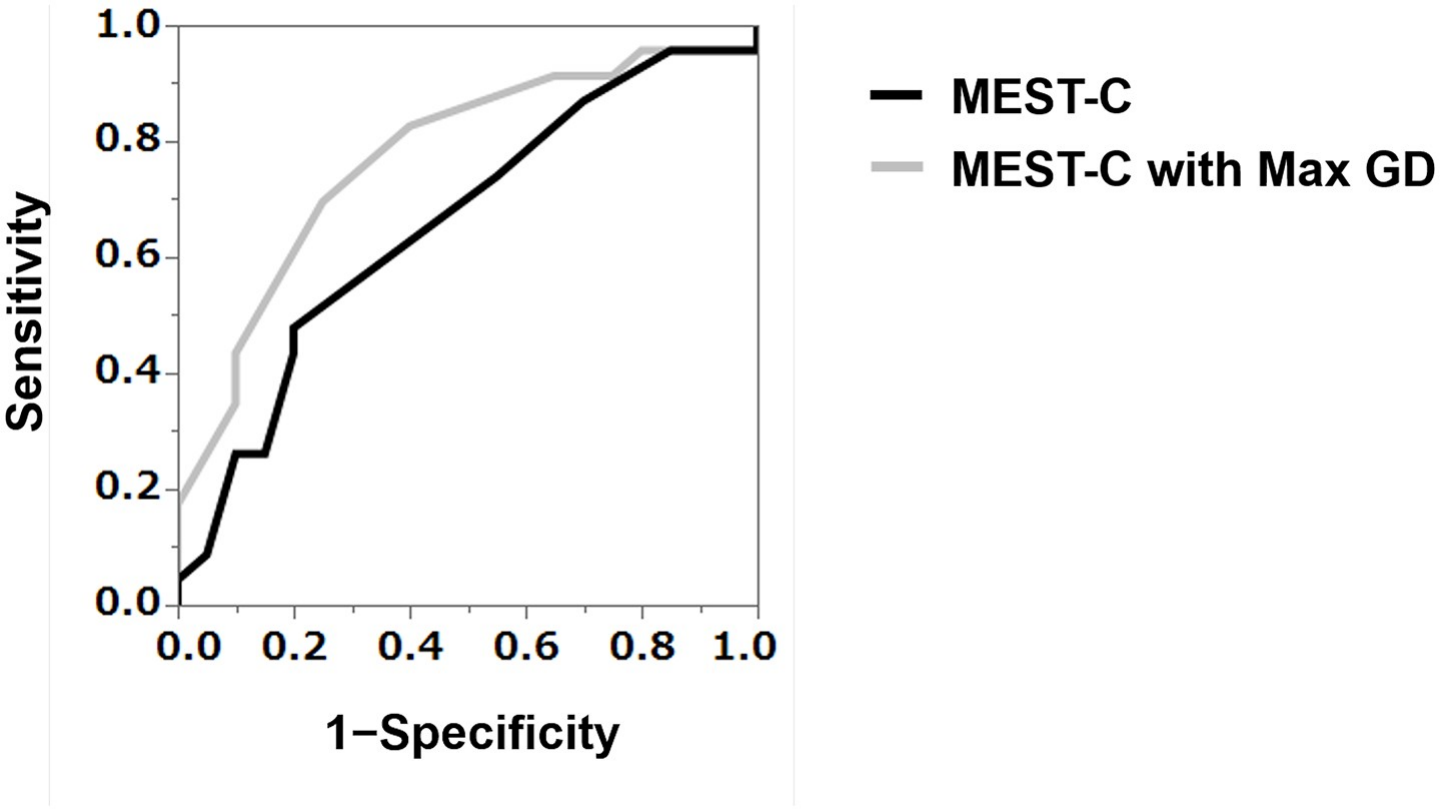
Receiver operating characteristic curves and the C-statistic (area under the curve) for the models predicting the risk of a ≥50% eGFR or ESRD using the Oxford (MEST-C) score with or without a maximal glomerular diameter (Max GD) ≥ 242.3 μm. Adding a Max GD ≥ 242.3 μm to the MEST-C score significantly improved discrimination regarding the renal outcome, as measured by the change in the C-statistic from 0.657 to 0.772. eGFR: estimated glomerular filtration rate; ESRD: end-stage renal disease; Max GD: maximal glomerular diameter.

To examine prognostic values of Max GD, we made two pathological models of IgAN consisting of MEST-C either with or without high-Max GD; we examined 1) the description ability of the model by C-statistic and 2) the goodness of fit of the model by pseudo-*R*^2^ and AICc. Addition of Max GD ≥ 242.3 μm to MEST-C scores improved the prediction of the renal outcome compared to using MEST-C scores alone. There was significant improvement in the ability to discriminate between those who did or did not experience the negative renal outcome after biopsy as measured by the change in C-statistic (0.657 up to 0.772) (Fig 2). There was also an increase in pseudo-*R*^2^ by 0.112 (0.033 vs. 0.145) and a reduction in AICc by 3.8 (71.8 down to 68.0), demonstrating better model fit.

### Time series changes in pseudo-*R*^2^ regarding prognostic efficacy

**Fig 3:**
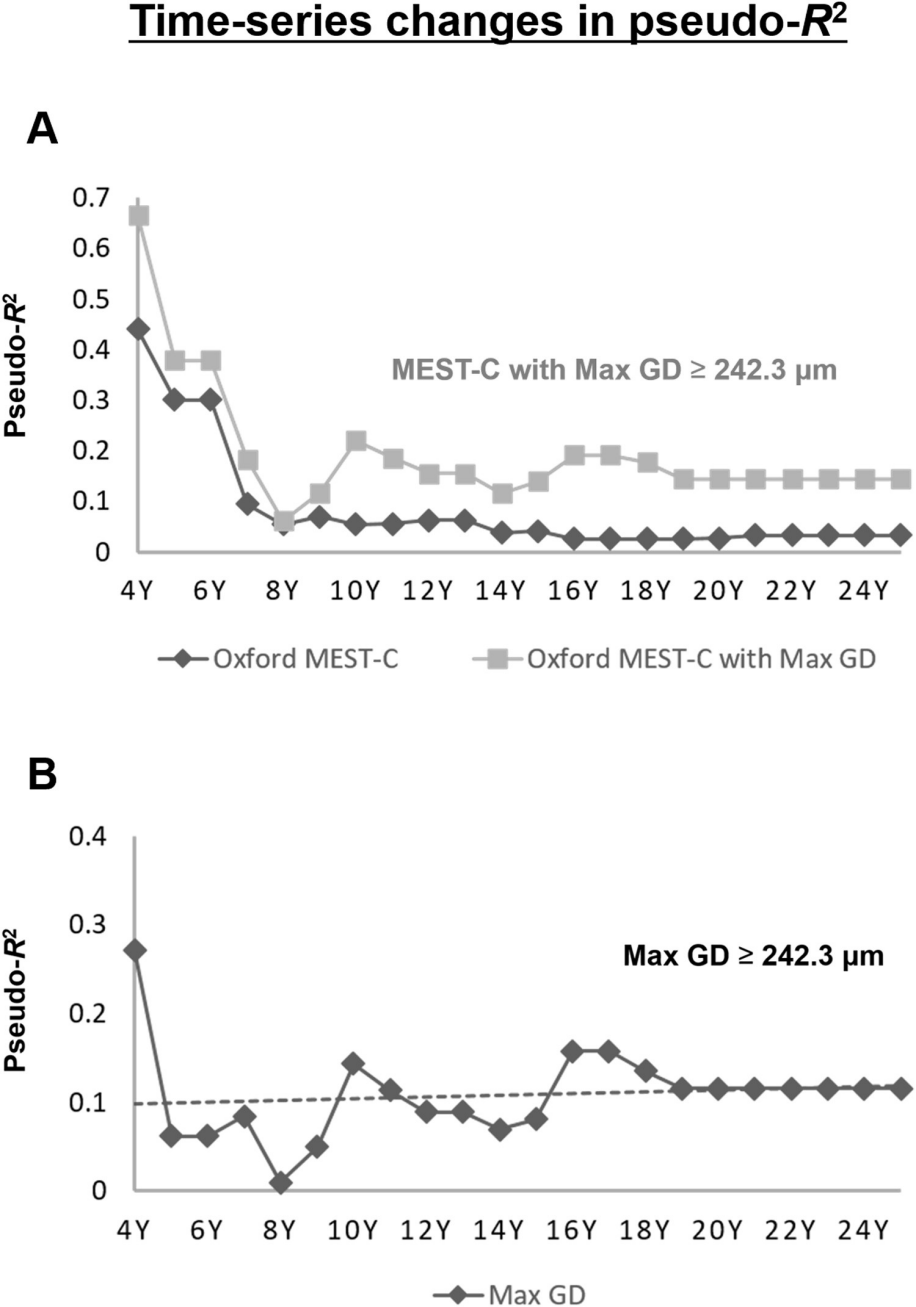
(A) Time-series changes in the pseudo-*R*^2^ values of the prognostic efficacy in relation to the renal outcome. The lower line represents the time-series changes in the pseudo-R^2^ values of the Oxford mesangial (M) and endocapillary (E) hypercellularity, segmental sclerosis (S), interstitial fibrosis/tubular atrophy (T), and crescents (C) (MEST-C) score and the upper line shows the time-series changes in the pseudo-*R*^2^ values of the Oxford MEST-C score with a maximal glomerular diameter (Max GD) ≥ 242.3 μm. Adding the Max GD improved the model’s short-term renal prognostic ability to about 1.5-fold for patients with IgAN 4 years after kidney biopsy, and the model’s long-term renal prognostic ability to more than 3-fold for patients with IgAN at the end of the study. Max GD: maximal glomerular diameter; eGFR: estimated glomerular filtration rate; ESRD: end-stage renal disease. (B) Time-series changes in the pseudo-*R*^2^ values for prognostic efficacy in relation to the renal outcome: Max GD. Although the prognostic potential of the Max GD tended to decrease after 4 years, it rose the predictive power with respect to renal prognosis after 8 years and sustained to the end of the study. Dotted line marks least-squares regression line. Max GD: maximal glomerular diameter.

Furthermore, to elucidate time series changes regarding renal prognostic ability of kidney pathological factors, we made further examination regarding *R*^2^ of Max GD, MEST-C score, and the sum of the MEST-C score with or without Max GD (Table 3, Fig 3). As shown in Fig 3A and Table 3, the values of the pseudo-*R*^2^ of the Oxford MEST-C were highest 4 years after kidney biopsy (0.4410), gradually fell until 8 years after kidney biopsy (0.0548), and then remained almost the same through the end of the follow-up period. The values of the pseudo-*R*^2^ of the model of ‘Max GD ≥ 242.3 μm with Oxford MEST-C’ showed the highest value of 0.6658 at the 4-years-post-kidney biopsy timepoint, fell to 0.0616 at the 8-years-post-kidney biopsy timepoint, and then rose to the value of 0.1449 at the end of the follow-up period. The values of the pseudo-*R*^2^ of the model of ‘Max GD ≥ 242.3μm with Oxford MEST-C’ was higher than those of ‘Oxford MEST-C’ at all the follow-up timepoints, demonstrating better model fit even in time series follow-up. As shown in Fig 3B and Table 3, the values of the pseudo-*R*^2^ of ‘Max GD ≥ 242.3μm’ showed a highest value of 0.2718 at the 4-years-post-kidney biopsy, fell to 0.0101 at the 8-years-post-kidney biopsy, and then rose to the value of 0.1156 at the end of the follow-up period.

**Table 3:** Time series changes in pseudo-R^2^ regarding prognostic efficacy for renal outcome: Max GD and Oxford MEST-C score.

	Max GD ≥ 242.3 μm	Oxford MEST-C	Oxford MEST-C with Max GD ≥ 242.3 μm
4Y	0.2718	0.4410	0.6658
5Y	0.0622	0.3015	0.3795
6Y	0.0622	0.3015	0.3795
7Y	0.0848	0.0945	0.1835
8Y	0.0101	0.0548	0.0616
9Y	0.0498	0.0697	0.1174
10Y	0.1437	0.0541	0.2209
11Y	0.1138	0.0561	0.1859
12Y	0.0896	0.0628	0.1552
13Y	0.0896	0.0628	0.1552
14Y	0.0696	0.0375	0.1166
15Y	0.0808	0.0411	0.1400
16Y	0.1579	0.0259	0.1916
17Y	0.1579	0.0259	0.1916
18Y	0.1355	0.0259	0.1775
19Y	0.1156	0.0259	0.1449
20Y	0.1156	0.0276	0.1449
21Y	0.1156	0.0333	0.1449
22Y	0.1156	0.0333	0.1449
23Y	0.1156	0.0333	0.1449
24Y	0.1156	0.0333	0.1449
End	0.1156	0.0333	0.1449

Abbreviations: Max GD, maximal glomerular diameter; Y, year: End, end of the study

## Discussion

To grasp the risk for ESRD over extended follow-up periods, lifetime risk is important knowledge for chronic kidney disease patients. IgAN is considered to have a variable progression rate due to differences in pathophysiological pathways. As mentioned in the previous report [2], some patients may not develop ESRD during the first year of follow-up, but they still have a substantial risk for ESRD over their lifetimes. Investigating the relationships of MEST-C scores or Max GD with long-term renal outcomes is clinically significant. In the present study, we found that long-term prediction was confirmed in the time series change of pseudo-*R*^2^ regarding renal prognosis. This finding could provide IgAN patients with important information about their disease progression.

### Significance of large renal corpuscles (glomerular hypertrophy)

Although it has been well-established that large renal corpuscles (glomerular hypertrophy) play a crucial role in the outcomes of kidney diseases in experimental models [16–19] and in humans [20–22], large glomerular size and renal corpuscle size as an indicator of renal prognosis have yet to be fully used in clinical settings. We propose that there is a pathological threshold of glomerular size which differentiates morbid glomerular hypertrophy from physiological glomerular hypertrophy [23], and we previously demonstrated poor renal prognosis of the high-Max GD group in patients with IgAN at the 10-year follow-up [7]. The present study demonstrated the poor renal prognosis of the high-Max GD group in patients with IgAN at the long-term follow-up. This result is important because histological risk factors are generally recognized to have weaker predictive power for long-term outcomes than clinical risk factors, such as persistent proteinuria or hypertension [24, 25]. The multivariate Cox regression analysis of the cohort indicated that large renal corpuscles (Max GD ≥ 242.3 μm) were associated with a ≥ 50% eGFR decrease or ESRD, confirmed by Kaplan-Meier analysis in age- and eGFR-matched models.

### Evaluation study of the Oxford classification of IgAN with Max GD

The C-statistic originated in diagnostic studies [12] and was generalized for survival data [13]. When comparing two models, an increase in C-statistic suggests improvement in discrimination. In the present study, significant improvement in discrimination regarding the renal outcome in C-statistic (0.657 up to 0.772) was achieved by adding Max GD ≥ 242.3 μm to MEST-C scores. Akaike Information Criterion is an estimator of the relative quality of statistical models, and the AICc was generalized to allow for small sample sizes [15], with a reduction in AICc suggesting better model fit. When Max GD was added to Oxford MEST-C scores, there was a reduction in AICc by 3.8 (71.8 down to 68.0), demonstrating better model fit. Pseudo-*R*^2^ is a measure of the explained variance that is valid in categorical regression models. Pseudo-R^2^ ranges from 0 to 1 (or 0% to 100%) with higher values indicating better model fit in logistic regressions [14]. In the present study, there was an increase in pseudo-*R*^2^ by 0.112 (0.033 vs. 0.145) when Max GD was added to Oxford MEST-C scores, suggesting better model fit. This result suggests that the score of the Oxford MEST-C combined with Max GD ≥ 242.3 μm explains 14.5% of renal outcome at the end of the present study, with long-term follow-up data. From the results of these three indices, we confirmed that the inclusion of the Max GD improves the renal long-term predictive prognostic ability in patients with IgAN.

Time series changes in pseudo-*R*^2^ regarding prognostic efficacy are rarely examined. In this study, we investigated the time series change of pseudo-*R*^2^, because long-term renal prognosis [2, 26] and time series changes regarding prognostic efficacy of risk factors are clinically relevant issues. The present study is the first to show the long-term follow-up time series change in pseudo-*R*^2^ values in relation to the prognostic abilities of renal pathological factors in IgAN patients. Though time series changes of prognostic abilities for renal outcome in IgAN patients were unknown, the values of the pseudo-R^2^s of MEST-C gradually fell over time, as we expected. The pseudo-*R*^2^ regarding the MEST-C fell from 0.4410 to 0.0548 between the 4- and 8-years-post-kidney biopsy timepoints, validating previous studies that found histological risk factors are worse predictors for the long-term outcome than clinical risk factors [24, 25]. To the contrary, although the values of the pseudo-*R*^2^ of ‘Max GD ≥ 242.3 μm’ also fell to the minimum of 0.0101 at the 8-years-post-kidney biopsy, it increased again to the value of 0.1156 at the end of the follow-up period. Assisted by the prognostic characteristics of ‘Max GD ≥ 242.3 μm’, the ‘MEST-C score combined with a Max GD ≥ 242.3 μm’ model explained 66.6% of renal outcomes 4 years after kidney biopsy, and 14.5% of renal outcomes at the end of the study. Adding Max GD improved the model’s short-term renal prognostic ability to 150.9% for patients with IgAN at the 4-year follow-up and improved the model’s long-term renal prognostic ability to 435.1% for patients with IgAN at the end of the study (calculations based on Table 3).

We propose that the reason for the rise of the pseudo-*R*2 value of ‘Max GD ≥ 242.3 μm’ is based on the multifactorial characteristics of glomerular hypertrophy. Glomerular hypertrophy has been well-reported by many studies to occur in various pathophysiological conditions and to play crucial roles in the outcomes in animal models of kidney disease, such as high-fructose diet-fed rat [27], high-fat diet-fed rhesus monkey [28], Otsuka Long-Evans Tokushima Fatty rat [18, 29], high protein diet-fed mouse [30], high cholesterol-fed rat [31], Dahl salt-sensitive rat [32], streptozotocin diabetic rat [33, 34], obese Zucker rat [35], human immunodeficiency virus type-1 transgenic mouse [36], SV40 transgenic mouse [37], growth hormone transgenic mouse [38], uninephrectomized rat received angiotensin II [17], and others [16, 19, 39–56]. In humans, glomerular hypertrophy is thought to reflect the activity of the original disease and the occurrence of renal damage caused by the presence of various metabolic risk factors such as obesity and diabetic mellitus [23]. In the present study, Max GD was affected by BMI and serum triglyceride and C3 levels, which were found to be higher in the high-Max GD group (Max GD ≥ 242.3 μm)(Table 1A). Although no patients were found to have diabetic nephropathy at the beginning of the study, it is important to consider diabetes or metabolic disorders when glomerular hypertrophy is detected on renal biopsy.

Generally, chronic kidney disease including IgAN is affected by multiple risk factors for disease progression [57, 58]. Therefore, it is extremely important to identify not only acute risk factors but also chronic risk factors for the acceleration of the kidney disease progression. Short-term renal prognoses are influenced by inflammation and lifestyle-related diseases, resulting in time-series decreases of the pseudo-*R*^2^ when patients were treated successfully. Long-term renal prognoses are influenced by irreversible lesions and lifestyle-related diseases, including obesity and atherosclerosis, generally resulting in time-series increases of the pseudo-*R*^2^ when patients were not treated successfully. The relationships between the Max GD pseudo-*R*^2^ value and short- and long-term renal prognoses suggest that the Max GD may represent a variety of pathological conditions, including immunological inflammation, lifestyle-related diseases, and irreversible damage.

In the present study, proteinuria and some Oxford lesions (mesangial hypercellularity, crescents, and segmental sclerosis) were not associated with the primary outcome (Table 2). At first glance, this seems discrepant with numerous published clinical and pathologic studies. However, the time series changes in pseudo-*R*^2^ values regarding prognostic efficacy provides elucidating information. As shown in Fig 3A and Table 3, although the value of the pseudo-*R*^2^ of the Oxford MEST-C was only 0.055 at 8 years after kidney biopsy, it remained greater than 0.3 until 6 years after kidney biopsy. Considering that most patients had active disease (with Oxford scores of M1 in 81.4%, E1 in 53.5%, S1 in 81.4%, T0 in 95.3%, C1 in 46.5%, and C2 in 9.3% of patients), and that 22 (51.2%) of the patients received steroid immunosuppression, the long-term renal prognoses might have been improved by successful treatment in the present study. Although the Oxford classification of IgAN [9, 10] was originally introduced to improve the individual risk prediction of IgAN progression, two major issues required resolution: the low renal prognostic ability of the Oxford classification [4, 26] and inconsistency in the renal prognostic power of each marker in the Oxford classification [59–62]. Differences regarding treatment, outcome measures, and patient selection criteria are thought to cause inconsistencies regarding the predictive values of the Oxford components. The assessment of the time series changes in pseudo-*R*^2^ values regarding renal prognostic efficacy might help resolve these inconsistencies.

### Limitations

The current results may have broader implications for patients with a variety of diseases due to our assessment of time series changes in pseudo-*R*^2^ of disease risk factors. However, our patient characteristics were determined only at baseline, and data during the follow-up period were not considered. Additionally, the sample size was relatively small, and further studies with a larger cohort are needed to confirm our findings. This study was observational in nature; thus, any observed associations do not prove causality.

## Conclusion

In conclusion, Max GD can be used as a long-term prognostic indicator for disease progression in IgAN patients. By adding Max GD ≥ 242.3 μm to Oxford MEST-C scores, there was significant improvement in the prediction of renal outcome in patients with IgAN at the long-term follow-up. Time series changes in pseudo-*R*^2^ regarding the Max GD provided highly suggestive information on renal progression of IgAN, which has multifactorial risk factors. We hope that the Max GD may be used as an early prognostic factor for the long-term outcomes of IgAN patients.

## Supporting information

S1 Fig Patient selection flow chart. (DOCX)

S1 Data Measurements of the covariates and definitions of comorbidities. (DOCX)

S1 Table Patient characteristics according to baseline Max GD levels (propensity score-matched cohort). (DOCX)

## References

[1] Knoop T, Vikse BE, Mwakimonga A, Leh S, Bjorneklett R. Long-term outcome in 145 patients with assumed benign immunoglobulin A nephropathy. Nephrol Dial Transplant. 2017;32(11):1841–50. Epub 2017/11/07. 10.1093/ndt/gfx242 . 29106593

[2] Coppo R, D'Arrigo G, Tripepi G, Russo ML, Roberts ISD, Bellur S, et al Is there long-term value of pathology scoring in immunoglobulin A nephropathy? A validation study of the Oxford Classification for IgA Nephropathy (VALIGA) update. Nephrol Dial Transplant. 2018 Epub 2018/11/13. 10.1093/ndt/gfy302 . 30418652

[3] Barbour S, Reich H. An update on predicting renal progression in IgA nephropathy. Current opinion in nephrology and hypertension. 2018;27(3):214–20. Epub 2018/02/13. 10.1097/MNH.0000000000000405 . 29432215

[4] Xie J, Lv J, Wang W, Li G, Liu Z, Chen H, et al Kidney Failure Risk Prediction Equations in IgA Nephropathy: A Multicenter Risk Assessment Study in Chinese Patients. American journal of kidney diseases: the official journal of the National Kidney Foundation. 2018;72(3):371–80. Epub 2018/03/21. 10.1053/j.ajkd.2018.01.043 . 29555434

[5] Chen T, Li X, Li Y, Xia E, Qin Y, Liang S, et al Prediction and Risk Stratification of Kidney Outcomes in IgA Nephropathy. American journal of kidney diseases: the official journal of the National Kidney Foundation. 2019 Epub 2019/04/30. 10.1053/j.ajkd.2019.02.016 . 31031086

[6] Trimarchi H, Barratt J, Cattran DC, Cook HT, Coppo R, Haas M, et al Oxford Classification of IgA nephropathy 2016: an update from the IgA Nephropathy Classification Working Group. Kidney international. 2017;91(5):1014–21. Epub 2017/03/28. 10.1016/j.kint.2017.02.003 . 28341274

[7] Kataoka H, Ohara M, Honda K, Mochizuki T, Nitta K. Maximal glomerular diameter as a 10-year prognostic indicator for IgA nephropathy. Nephrol Dial Transplant. 2011;26(12):3937–43. Epub 2011/03/24. 10.1093/ndt/gfr139 . 21427079

[8] Kataoka H, Ohara M, Honda K, Mochizuki T, Nitta K. Maximal glomerular diameter as a 10-year prognostic indicator for IgA nephropathy. Nephrology Dialysis Transplantation. 2011;26(12):3937–U674. 10.1093/ndt/gfr139 21427079

[9] Roberts IS, Cook HT, Troyanov S, Alpers CE, Amore A, Barratt J, et al The Oxford classification of IgA nephropathy: pathology definitions, correlations, and reproducibility. Kidney international. 2009;76(5):546–56. Epub 2009/07/03. 10.1038/ki.2009.168 . 19571790

[10] Cattran DC, Coppo R, Cook HT, Feehally J, Roberts ISD, Troyanov S, et al The Oxford classification of IgA nephropathy: rationale, clinicopathological correlations, and classification. Kidney international. 2009;76(5):534–45. 10.1038/ki.2009.243 19571791

[11] Lane PH, Steffes MW, Mauer SM. Estimation of glomerular volume: a comparison of four methods. Kidney international. 1992;41(4):1085–9. Epub 1992/04/01. 10.1038/ki.1992.165 . 1513090

[12] Hanley JA, McNeil BJ. The meaning and use of the area under a receiver operating characteristic (ROC) curve. Radiology. 1982;143(1):29–36. Epub 1982/04/01. 10.1148/radiology.143.1.7063747 . 7063747

[13] Harrell FE Jr., Califf RM, Pryor DB, Lee KL, Rosati RA. Evaluating the yield of medical tests. Jama. 1982;247(18):2543–6. Epub 1982/05/14. . 7069920

[14] Hauber AB, Gonzalez JM, Groothuis-Oudshoorn CG, Prior T, Marshall DA, Cunningham C, et al Statistical Methods for the Analysis of Discrete Choice Experiments: A Report of the ISPOR Conjoint Analysis Good Research Practices Task Force. Value Health. 2016;19(4):300–15. Epub 2016/06/22. 10.1016/j.jval.2016.04.004 . 27325321

[15] Hurvich CM, Tsai CL. Model selection for extended quasi-likelihood models in small samples. Biometrics. 1995;51(3):1077–84. Epub 1995/09/01. . 7548692

[16] Fries JW, Sandstrom DJ, Meyer TW, Rennke HG. Glomerular hypertrophy and epithelial cell injury modulate progressive glomerulosclerosis in the rat. Laboratory investigation; a journal of technical methods and pathology. 1989;60(2):205–18. Epub 1989/02/01. . 2915515

[17] Miller PL, Rennke HG, Meyer TW. Glomerular hypertrophy accelerates hypertensive glomerular injury in rats. The American journal of physiology. 1991;261(3 Pt 2):F459–65. Epub 1991/09/01. 10.1152/ajprenal.1991.261.3.F459 . 1887907

[18] Toyota E, Ogasawara Y, Fujimoto K, Kajita T, Shigeto F, Asano T, et al Global heterogeneity of glomerular volume distribution in early diabetic nephropathy. Kidney international. 2004;66(2):855–61. 10.1111/j.1523-1755.2004.00816.x 15253743

[19] Cahill MM, Ryan GB, Bertram JF. Biphasic glomerular hypertrophy in rats administered puromycin aminonucleoside. Kidney international. 1996;50(3):768–75. Epub 1996/09/01. 10.1038/ki.1996.375 . 8872950

[20] Fogo A, Hawkins EP, Berry PL, Glick AD, Chiang ML, MacDonell RC Jr., et al Glomerular hypertrophy in minimal change disease predicts subsequent progression to focal glomerular sclerosis. Kidney international. 1990;38(1):115–23. Epub 1990/07/01. 10.1038/ki.1990.175 . 2385079

[21] Osterby R, Gundersen HJ. Glomerular size and structure in diabetes mellitus. I. Early abnormalities. Diabetologia. 1975;11(3):225–9. Epub 1975/06/01. 10.1007/bf00422326 . 1149955

[22] Zerbini G, Bonfanti R, Meschi F, Bognetti E, Paesano PL, Gianolli L, et al Persistent renal hypertrophy and faster decline of glomerular filtration rate precede the development of microalbuminuria in type 1 diabetes. Diabetes. 2006;55(9):2620–5. 10.2337/db06-0592 16936212

[23] Kataoka H, Mochizuki T, Nitta K. Large Renal Corpuscle: Clinical Significance of Evaluation of the Largest Renal Corpuscle in Kidney Biopsy Specimens. Contrib Nephrol. 2018;195:20–30. Epub 2018/05/08. 10.1159/000486931 . 29734147

[24] Donadio JV, Bergstralh EJ, Grande JP, Rademcher DM. Proteinuria patterns and their association with subsequent end-stage renal disease in IgA nephropathy. Nephrology Dialysis Transplantation. 2002;17(7):1197–203. 10.1093/ndt/17.7.1197 12105241

[25] Goto M, Kawamura T, Wakai K, Ando M, Endoh M, Tomino Y. Risk stratification for progression of IgA nephropathy using a decision tree induction algorithm. Nephrology Dialysis Transplantation. 2009;24(4):1242–7. 10.1093/ndt/gfn610 19017674 PMC2658733

[26] Barbour SJ, Espino-Hernandez G, Reich HN, Coppo R, Roberts IS, Feehally J, et al The MEST score provides earlier risk prediction in lgA nephropathy. Kidney international. 2016;89(1):167–75. Epub 2016/01/14. 10.1038/ki.2015.322 . 26759049

[27] Cosenzi A, Bernobich E, Plazzotta N, Seculin P, Bellini G. Bosentan reduces blood pressure and the target-organ damage induced by a high-fructose diet in rats. J Hypertens. 1999;17(12 Pt 2):1843–8. Epub 2000/03/07. 10.1097/00004872-199917121-00010 . 10703878

[28] Cusumano AM, Bodkin NL, Hansen BC, Iotti R, Owens J, Klotman PE, et al Glomerular hypertrophy is associated with hyperinsulinemia and precedes overt diabetes in aging rhesus monkeys. Am J Kidney Dis. 2002;40(5):1075–85. Epub 2002/10/31. 10.1053/ajkd.2002.36348 . 12407654

[29] Kim S, Wanibuchi H, Hamaguchi A, Miura K, Yamanaka S, Iwao H. Angiotensin blockade improves cardiac and renal complications of type II diabetic rats. Hypertension. 1997;30(5):1054–61. Epub 1997/11/22. 10.1161/01.hyp.30.5.1054 . 9369255

[30] Schrijvers BF, Rasch R, Tilton RG, Flyvbjerg A. High protein-induced glomerular hypertrophy is vascular endothelial growth factor-dependent. Kidney Int. 2002;61(5):1600–4. Epub 2002/04/23. 10.1046/j.1523-1755.2002.00310.x . 11967009

[31] Guijarro C, Kasiske BL, Kim Y, O'Donnell MP, Lee HS, Keane WF. Early glomerular changes in rats with dietary-induced hypercholesterolemia. Am J Kidney Dis. 1995;26(1):152–61. Epub 1995/07/01. 10.1016/0272-6386(95)90169-8 . 7611247

[32] Bledsoe G, Shen B, Yao Y, Zhang JJ, Chao L, Chao J. Reversal of renal fibrosis, inflammation, and glomerular hypertrophy by kallikrein gene delivery. Hum Gene Ther. 2006;17(5):545–55. Epub 2006/05/24. 10.1089/hum.2006.17.545 . 16716111

[33] Osterby R, Gundersen HJ. Fast accumulation of basement membrane material and the rate of morphological changes in acute experimental diabetic glomerular hypertrophy. Diabetologia. 1980;18(6):493–500. Epub 1980/06/01. 10.1007/bf00261706 . 7418958

[34] Usui H, Shikata K, Matsuda M, Okada S, Ogawa D, Yamashita T, et al HMG-CoA reductase inhibitor ameliorates diabetic nephropathy by its pleiotropic effects in rats. Nephrol Dial Transplant. 2003;18(2):265–72. Epub 2003/01/25. 10.1093/ndt/18.2.265 . 12543879

[35] Michel O, Heudes D, Lamarre I, Masurier C, Lavau M, Bariety J, et al Reduction of insulin and triglycerides delays glomerulosclerosis in obese Zucker rats. Kidney Int. 1997;52(6):1532–42. Epub 1998/01/04. 10.1038/ki.1997.483 . 9407498

[36] Bruggeman LA, Dikman S, Meng C, Quaggin SE, Coffman TM, Klotman PE. Nephropathy in human immunodeficiency virus-1 transgenic mice is due to renal transgene expression. J Clin Invest. 1997;100(1):84–92. Epub 1997/07/01. 10.1172/JCI119525 . 9202060 PMC508168

[37] MacKay K, Striker LJ, Stauffer JW, Agodoa LY, Striker GE. Relationship of glomerular hypertrophy and sclerosis: studies in SV40 transgenic mice. Kidney Int. 1990;37(2):741–8. Epub 1990/02/01. 10.1038/ki.1990.41 . 2308261

[38] Doi T, Striker LJ, Gibson CC, Agodoa LY, Brinster RL, Striker GE. Glomerular lesions in mice transgenic for growth hormone and insulinlike growth factor-I. I. Relationship between increased glomerular size and mesangial sclerosis. Am J Pathol. 1990;137(3):541–52. Epub 1990/09/01. . 2399934 PMC1877515

[39] Shimamura T, Morrison AB. A progressive glomerulosclerosis occurring in partial five-sixths nephrectomized rats. Am J Pathol. 1975;79(1):95–106. Epub 1975/04/01. . 164779 PMC1913032

[40] Olivetti G, Anversa P, Rigamonti W, Vitali-Mazza L, Loud AV. Morphometry of the renal corpuscle during normal postnatal growth and compensatory hypertrophy. A light microscope study. J Cell Biol. 1977;75(2 Pt 1):573–85. Epub 1977/11/01. 10.1083/jcb.75.2.573 . 264124 PMC2109942

[41] Nagata M, Scharer K, Kriz W. Glomerular damage after uninephrectomy in young rats. I. Hypertrophy and distortion of capillary architecture. Kidney Int. 1992;42(1):136–47. Epub 1992/07/01. 10.1038/ki.1992.271 . 1635343

[42] Rayner HC, Ward L, Walls J. Cholesterol feeding following unilateral nephrectomy in the rat leads to glomerular hypertrophy. Nephron. 1991;57(4):453–9. Epub 1991/01/01. 10.1159/000186349 . 2046829

[43] Rodriguez-Lopez AM, Flores O, Arevalo MA, Lopez-Novoa JM. Glomerular cell proliferation and apoptosis in uninephrectomized spontaneously hypertensive rats. Kidney Int Suppl. 1998;68:S36–40. Epub 1998/12/05. 10.1046/j.1523-1755.1998.06810.x . 9839281

[44] Wolf G, Schroeder R, Thaiss F, Ziyadeh FN, Helmchen U, Stahl RA. Glomerular expression of p27Kip1 in diabetic db/db mouse: role of hyperglycemia. Kidney Int. 1998;53(4):869–79. Epub 1998/04/29. 10.1111/j.1523-1755.1998.00829.x . 9551393

[45] Phillips AO, Baboolal K, Riley S, Grone H, Janssen U, Steadman R, et al Association of prolonged hyperglycemia with glomerular hypertrophy and renal basement membrane thickening in the Goto Kakizaki model of non-insulin-dependent diabetes mellitus. Am J Kidney Dis. 2001;37(2):400–10. Epub 2001/02/07. 10.1053/ajkd.2001.21322 . 11157383

[46] Nakagawa T, Mazzali M, Kang DH, Kanellis J, Watanabe S, Sanchez-Lozada LG, et al Hyperuricemia causes glomerular hypertrophy in the rat. Am J Nephrol. 2003;23(1):2–7. Epub 2002/10/10. 10.1159/000066303 . 12373074

[47] Wei P, Lane PH, Lane JT, Padanilam BJ, Sansom SC. Glomerular structural and functional changes in a high-fat diet mouse model of early-stage Type 2 diabetes. Diabetologia. 2004;47(9):1541–9. Epub 2004/09/01. 10.1007/s00125-004-1489-1 . 15338127

[48] Wiggins JE, Goyal M, Sanden SK, Wharram BL, Shedden KA, Misek DE, et al Podocyte hypertrophy, "adaptation," and "decompensation" associated with glomerular enlargement and glomerulosclerosis in the aging rat: prevention by calorie restriction. J Am Soc Nephrol. 16. United States 2005 p. 2953–66. 10.1681/ASN.2005050488 16120818

[49] Suzuki H, Tokuriki T, Saito K, Hishida A, Suzuki K. Glomerular hyperfiltration and hypertrophy in the rat hypoplastic kidney as a model of oligomeganephronic disease. Nephrol Dial Transplant. 2005;20(7):1362–9. Epub 2005/05/05. 10.1093/ndt/gfh782 . 15870220

[50] Dworkin LD, Hostetter TH, Rennke HG, Brenner BM. Hemodynamic basis for glomerular injury in rats with desoxycorticosterone-salt hypertension. J Clin Invest. 1984;73(5):1448–61. Epub 1984/05/01. 10.1172/JCI111349 . 6715546 PMC425168

[51] Uemasu J, Godai K, Tokumoto A, Kawasaki H. Reduced glomerular hypertrophy by somatostatin analog, SMS 201–995, in the subtotal nephrectomized rats fed high-protein meals. J Pharmacol Exp Ther. 1992;260(2):505–8. Epub 1992/02/01. . 1738100

[52] Gu JW, Bailey AP, Tan W, Shparago M, Young E. Long-term High Salt Diet Causes Hypertension and Decreases Renal Expression of Vascular Endothelial Growth Factor in Sprague-Dawley Rats. J Am Soc Hypertens. 2008;2(4):275–85. Epub 2009/01/06. 10.1016/j.jash.2008.03.001 . 19122855 PMC2598434

[53] Ijpelaar DH, Schulz A, Koop K, Schlesener M, Bruijn JA, Kerjaschki D, et al Glomerular hypertrophy precedes albuminuria and segmental loss of podoplanin in podocytes in Munich-Wistar-Fromter rats. Am J Physiol Renal Physiol. 2008;294(4):F758–67. Epub 2008/01/18. 10.1152/ajprenal.00457.2007 . 18199599

[54] Rosenmann E, Teitelbaum A, Cohen AM. Nephropathy in sucrose-fed rats. Electron and light microscopic studies. Diabetes. 1971;20(12):803–10. Epub 1971/12/01. 10.2337/diab.20.12.803 . 4330451

[55] Li L, Zhao Z, Xia J, Xin L, Chen Y, Yang S, et al A Long-Term High-Fat/High-Sucrose Diet Promotes Kidney Lipid Deposition and Causes Apoptosis and Glomerular Hypertrophy in Bama Minipigs. PLoS One. 2015;10(11):e0142884 Epub 2015/11/17. 10.1371/journal.pone.0142884 . 26571016 PMC4646641

[56] Roncal-Jimenez CA, Ishimoto T, Lanaspa MA, Milagres T, Hernando AA, Jensen T, et al Aging-associated renal disease in mice is fructokinase dependent. Am J Physiol Renal Physiol. 2016;311(4):F722–f30. Epub 2016/07/29. 10.1152/ajprenal.00306.2016 . 27465991 PMC5142232

[57] Nenov VD, Taal MW, Sakharova OV, Brenner BM. Multi-hit nature of chronic renal disease. Current opinion in nephrology and hypertension. 2000;9(2):85–97. 10.1097/00041552-200003000-00001 10757212

[58] Taal MW, Brenner BM. Predicting initiation and progression of chronic kidney disease: Developing renal risk scores. Kidney international. 2006;70(10):1694–705. 10.1038/sj.ki.5001794 16969387

[59] Coppo R, Troyanov S, Bellur S, Cattran D, Cook HT, Feehally J, et al Validation of the Oxford classification of IgA nephropathy in cohorts with different presentations and treatments. Kidney international. 2014;86(4):828–36. Epub 2014/04/04. 10.1038/ki.2014.63 . 24694989 PMC4184028

[60] Herzenberg AM, Fogo AB, Reich HN, Troyanov S, Bavbek N, Massat AE, et al Validation of the Oxford classification of IgA nephropathy. Kidney international. 2011;80(3):310–7. Epub 2011/05/06. 10.1038/ki.2011.126 . 21544062

[61] Moriyama T, Nakayama K, Iwasaki C, Ochi A, Tsuruta Y, Itabashi M, et al Severity of nephrotic IgA nephropathy according to the Oxford classification. International urology and nephrology. 2012;44(4):1177–84. 10.1007/s11255-011-0109-5 22231129

[62] Shi SF, Wang SX, Jiang L, Lv JG, Liu LJ, Chen YQ, et al Pathologic Predictors of Renal Outcome and Therapeutic Efficacy in IgA Nephropathy: Validation of the Oxford Classification. Clinical Journal of the American Society of Nephrology. 2011;6(9):2175–84. 10.2215/CJN.11521210 21852672 PMC3358999

