# Supplementary figures and images for: Time series changes in pseudo-R2 values regarding maximum glomerular diameter and the Oxford MEST-C score in patients with IgA nephropathy: A long-term follow-up study
Source: PLoS One. 2020 May 7;15(5):e0232885. doi: 10.1371/journal.pone.0232885 (PMC7205238; doi:10.1371/journal.pone.0232885)

**
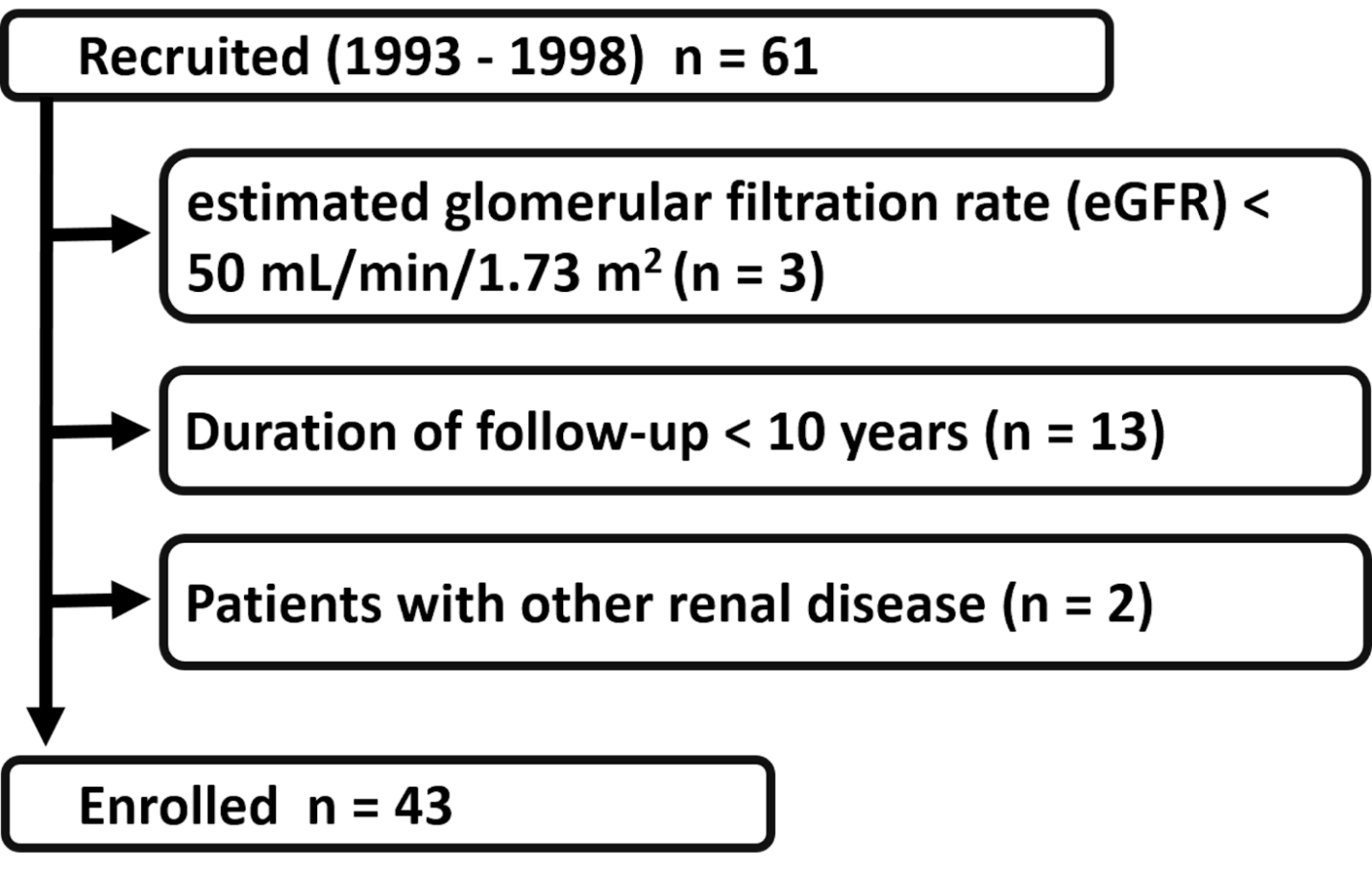
**

Supplement: S1 Fig — (DOCX) [file pone.0232885.s001.docx]
