# Supplementary material for: Time series changes in pseudo-R2 values regarding maximum glomerular diameter and the Oxford MEST-C score in patients with IgA nephropathy: A long-term follow-up study
Source: PLoS One. 2020 May 7;15(5):e0232885. doi: 10.1371/journal.pone.0232885 (PMC7205238; doi:10.1371/journal.pone.0232885)
Supplement: S1 Data — (DOCX) [file pone.0232885.s002.docx]

**Web appendix to “Time series changes in pseudo-R^2^ values regarding maximum glomerular diameter and the Oxford MEST-C score in patients with IgA nephropathy: a long-term follow-up study”**

This appendix provides further methodological detail and results for the main paper.

**Patient selection flow chart.** From 61 initially patients screened, 3 whose estimated glomerular filtration rate (eGFR) < 50 mL/min/1.73 m^2^, 13 whose duration of follow-up < 10 years, and 2 patients with other renal disease were excluded; the remaining 43 patients were enrolled in this study

**Supplementary Methods**

**Measurements of the covariates**

The baseline parameters assessed in this study were age and sex, the mean blood pressure (MBP),　the estimated glomerular filtration rate (eGFR), and the hemoglobin, serum albumin, creatinine, blood urea nitrogen, uric acid, total cholesterol, triglyceride (TG), immunoglobulin G (IgG), immunoglobulin A (IgA), immunoglobulin M (IgM), 50% hemolytic complement activity (CH50), complement component 3 (C3), C4, complement component 4 (C4), and hemoglobin A1c levels, and urinary protein excretion (U-Prot), urinary red blood cells (U-RBC). All of the biochemical analyses were performed on samples obtained after overnight fasts. The serum creatinine levels were measured enzymatically. The eGFR for Japanese patients was calculated using a previously described formula [1].

**Definition of comorbidities**

The impacts of concomitant agent use and comorbidities at baselin were also assessed [2]. The comorbidities were recorded as positive according to the criteria described next. Hypertension was defined as BP ≥140/90 mmHg and/or taking an antihypertensive agent. Hypercholesterolemia was defined as a serum TC level ≥220 mg/dL and/or taking an antidyslipidemic agent. Hypertriglyceridemia was defined as serum TG level ≥ 150 mg/dL and/or taking an antidyslipidemic agent. Hyperuricemia was defined as serum UA level ≥ 7.0 mg/dL and/or taking an antihyperuricemic agent. We also assessed initial treatment regimens, defined as treatment performed during the first year after renal biopsy.

**Supplementary References**

1. Matsuo S, Imai E, Horio M*, et al.* Revised Equations for Estimated GFR From Serum Creatinine in Japan. American Journal of Kidney Diseases 2009;53(6):982-992

2. Ording AG, Sorensen HT. Concepts of comorbidities, multiple morbidities, complications, and their clinical epidemiologic analogs. Clin Epidemiol 2013;5:199-203
