# Supplementary material for: Time series changes in pseudo-R2 values regarding maximum glomerular diameter and the Oxford MEST-C score in patients with IgA nephropathy: A long-term follow-up study
Source: PLoS One. 2020 May 7;15(5):e0232885. doi: 10.1371/journal.pone.0232885 (PMC7205238; doi:10.1371/journal.pone.0232885)
Supplement: S1 Table — (DOCX) [file pone.0232885.s003.docx]

**Table S1A. Patient characteristics according to baseline Max GD levels**

**(Table S1A. Clinical and laboratory findings; Propensity score matched cohort, n = 24)**

| Variables | Overall | Max GD  ≥ 242.3 μm | Max GD  < 242.3 μm | *P*- value | Standardized  Differences |
| --- | --- | --- | --- | --- | --- |
|  | n = 24 | n = 12 | n = 12 |  |  |
| *Clinical Findings* |  |  |  |  |  |
| Age (years) | 42.2 ± 8.7 | 40.7 ± 10.2 | 43.7 ± 7.0 | 0.4179 | 0.343 |
| Sex [Men; n (%)] | 17 (70.8) | 8 (66.7) | 9 (75.0) | 1.0000 | 0.183 |
| BMI (kg/m^2^) | 26.8 ± 3.6 | 27.7 ± 4.0 | 25.9 ± 2.9 | 0.2252 | 0.515 |
| SBP (mmHg) | 144.2 ± 25.3 | 142.3 ± 22.6 | 146.2 ± 28.6 | 0.7129 | 0.151 |
| DBP (mmHg) | 84.3 ± 17.0 | 83.2 ± 14.5 | 85.5 ± 19.7 | 0.7444 | 0.133 |
| *Laboratory Findings* |  |  |  |  |  |
| Total Protein (g/dL) | 6.50 ± 0.64 | 6.75 ± 0.52 | 6.25 ± 0.67 | 0.0531 | 0.834 |
| Serum Albumin (g/dL) | 3.75 ± 0.43 | 3.89 ± 0.43 | 3.60 ± 0.40 | 0.0984 | 0.698 |
| Blood Urea Nitrogen (mg/dL) | 14.8 ± 3.0 | 15.3 ± 2.9 | 14.2 ± 3.1 | 0.3492 | 0.366 |
| Serum Creatinine (mg/dL) | 0.90 ± 0.21 | 0.90 ± 0.23 | 0.89 ± 0.19 | 0.9249 | 0.047 |
| eGFR (mL/min/1.73m^2^) | 72.2 ± 12.3 | 72.1 ± 12.9 | 72.3 ± 12.3 | 0.9720 | 0.016 |
| Uric Acid (mg/dL) | 6.18 ± 1.77 | 6.04 ± 1.61 | 6.31 ± 1.98 | 0.7208 | 0.150 |
| Total Cholesterol (mg/dL) | 202.3 ± 47.9 | 195.6 ± 21.8 | 209.0 ± 64.9 | 0.5046 | 0.277 |
| Triglyceride (mg/dL) | 188.3 ± 148.6 | 213.6 ± 203.5 | 163.1 ± 58.4 | 0.4174 | 0.337 |
| Hemoglobin A1c (NGSP) (%) | 5.34 ± 0.41 | 5.50 ± 0.28 | 5.22 ± 0.47 | 0.1560 | 0.724 |
| IgG (mg/dL) | 1169.0 ± 351.0 | 1164.6 ± 451.4 | 1173.5 ± 231.9 | 0.9520 | 0.025 |
| IgA (mg/dL) | 362.1 ± 149.0 | 372.1 ± 175.9 | 352.1 ± 123.6 | 0.7499 | 0.132 |
| IgM (mg/dL) | 164.3 ± 99.0 | 159.5 ± 87.0 | 169.2 ± 113.5 | 0.8172 | 0.096 |
| CH50 (mg/dL) | 41.0 ± 5.2 | 40.4 ± 6.0 | 41.8 ± 4.3 | 0.5817 | 0.268 |
| C3c (mg/dL) | 90.9 ± 20.3 | 101.5 ± 21.6 | 80.2 ± 12.2 | 0.0069 | 1.214 |
| C4 (mg/dL) | 37.7 ± 11.3 | 39.1 ± 12.1 | 36.4 ± 10.8 | 0.5695 | 0.235 |
| IgA/C3 ratio | 4.14 ± 1.88 | 3.71 ± 1.70 | 4.58 ± 2.03 | 0.2636 | 0.465 |
| U-Prot (g/day) | 1.6 (0.0–7.0) | 1.7 (0.6–2.6) | 1.5 (0.0–7.0) | 1.0000 | 0.289 |
| U-RBC (counts/HF) | 10 (0–200) | 5.5 (0–100) | 10 (0–200) | 0.6181 | 0.184 |
| *Initial treatments* |  |  |  |  |  |
| Corticosteroids [n (%)] | 15 (62.5) | 6 (50.0) | 9 (75.0) | 0.4003 | 0.535 |
| Tonsillectomy [n (%)] | 0 (0.0) | 0 (0.0) | 0 (0.0) | NA | NA |
| Immunosuppressants [n (%)] | 0 (0.0) | 0 (0.0) | 0 (0.0) | NA- | NA |
| *Concomitant drugs* |  |  |  |  |  |
| Antihypertensive agents [n (%)] | 17 (70.8) | 8 (66.7) | 9 (75.0) | 1.0000 | 0.183 |
| ARB and or ACEI [n (%)] | 15 (62.5) | 7 (58.3) | 8 (66.7) | 1.0000 | 0.174 |
| Others [n (%)] | 8 (33.3) | 4 (33.3) | 4 (33.3) | 1.0000 | 0.000 |
| Anti-platelet agents | 23 (95.8) | 11 (91.7) | 12 (100.0) | 1.0000 | 0.425 |
| Anti-coagulation | 8 (33.3) | 2 (16.7) | 6 (50.0) | 0.1930 | 0.755 |
| Antidyslipidemic agents [n (%)] | 1 (4.2) | 1 (8.3) | 0 (0.0) | 1.0000 | 0.425 |
| Antihyperuricemic agents [n (%)] | 5 (20.8) | 2 (16.7) | 3 (25.0) | 1.0000 | 0.205 |
| *Comorbidities* |  |  |  |  |  |
| Hypertension [n (%)] | 19 (79.2) | 9 (75.0) | 10 (83.3) | 1.0000 | 0.205 |
| Hyperuricemia [n (%)] | 11 (45.8) | 6 (50.0) | 5 (41.7) | 1.0000 | 0.167 |
| Hypertriglyceridemia [n (%)] | 13 (54.2) | 6 (50.0) | 7 (58.3) | 1.0000 | 0.167 |
| Hypercholesterolemia [n (%)] | 7 (29.2) | 3 (25.0) | 4 (33.3) | 1.0000 | 0.183 |

**(Table S1B. Histological findings; Propensity score matched cohort, n = 24)**

| Variables | Overall | Max GD  ≥ 242.3 μm | Max GD  < 242.3 μm | *P*- value | Standardized  Differences |
| --- | --- | --- | --- | --- | --- |
|  | n = 24 | n = 12 | n = 12 |  |  |
| Number of glomeruli | 12 (5–24) | 11 (5–24) | 14 (5–18) | 0.4174 | 0.168 |
| Global sclerosis (%) | 12.5 (0.0–50.0) | 11.8 (0.0–36.4) | 13.4 (0.0–50.0) | 0.5423 | 0.401 |
| Segmental sclerosis (%) | 15.5 (0.0–55.6) | 15.5 (0.0–55.6) | 15.3 (0.0–42.9) | 0.6609 | 0.059 |
| Crescent (%) | 11.4 (0.0–33.3) | 5.6 (0.0–33.3) | 12.1 (0.0–28.6) | 0.4354 | 0.223 |
| Cellular or Fibro-Cellular (%) | 8.7 (0.0–28.6) | 5.6 (0.0–22.2) | 9.0 (0.0–28.6) | 0.4535 | 0.278 |
| Fibrous (%) | 0.0 (0.0–12.5) | 0.0 (0.0–12.5) | 0.0 (0.0–8.3) | 0.7327 | 0.253 |
| Mesangial cell proliferation (0-3) | 3 (1–3) | 3 (1–3) | 2.5 (1–3) | 0.3276 | 0.425 |
| Interstitial fibrosis (%) | 5.0 (0.0–30.0) | 5.0 (3.0–25.0) | 7.5 (0.0–30.0) | 0.4392 | 0.373 |
| Interstitial fibrosis (0-3) | 1 (0–2) | 1 (0–2) | 0.5 (0–2) | 0.4483 | 0.238 |
| Interstitial inflammation (%) | 5.0 (0.0–30.0) | 5.0 (0.0–25.0) | 2.5 (0.0–30.0) | 0.6543 | 0.129 |
| Arteriosclerosis (0-3) | 1 (0–2) | 1 (0–2) | 0.5 (0–2) | 0.2645 | 0.434 |
| Arteriolar hyalinosis (0-3) | 1 (0–3) | 1 (0–3) | 1 (0–2) | 0.0822 | 0.752 |
| MaxGD (µm) | 236.0 ± 22.9 | 253.9 ± 11.9 | 218.2 ± 16.2 | <0.0001 | 2.512 |
| MaxGA (µm) | 42211.6 ± 8485.0 | 48559.1 ± 5403.1 | 35864.2 ± 5782.5 | <0.0001 | 2.269 |
| *Oxford Classification* |  |  |  |  |  |
| M1 | 21 (87.5) | 10 (83.3) | 11 (91.7) | 1.0000 | 0.256 |
| E1 | 13 (54.2) | 7 (58.3) | 6 (50.0) | 1.0000 | 0.167 |
| S1 | 20 (83.3) | 9 (75.0) | 11 (91.7) | 0.5901 | 0.460 |
| T1 | 1 (4.2) | 0 (0.0) | 1 (8.3) | 1.0000 | 0.425 |
| T2 | 0 (0.0) | 0 (0.0) | 0 (0.0) | NA | NA |
| C1 | 13 (54.2) | 6 (50.0) | 7 (58.3) | 1.0000 | 0.167 |
| C2 | 1 (4.2) | 0 (0.0) | 1 (8.3) | 1.0000 | 0.425 |

Continuous variables are expressed as means ± standard deviation or median (minimum–maximum). Count data are expressed as n (%). Abbreviations: n, number; %, percentages; BMI, body mass index; SBP, systolic blood pressure; DBP, diastolic blood pressure; eGFR, estimated glomerular filtration rate; IgG, immunoglobulin G; IgA, immunoglobulin A; IgM, immunoglobulin M; CH50, 50% hemolytic complement activity; C3, complement component 3; C4, complement component 4; U-Prot, Urinary protein excretion; U-RBC, urinary red blood cells; ARB, angiotensin receptor blocker; ACEI, angiotensin-converting enzyme inhibitor; Max GD, maximal glomerular diameter; NA, not applicable; M, mesangial hypercellularity; E, endocapillary hypercellularity; S, segmental glomerulosclerosis; T, tubular atrophy/interstitial fibrosis; C, cellular/fibrocellular crescents
